# Orthographic relatedness and transposed-word effects in the grammatical decision task

**DOI:** 10.3758/s13414-021-02421-y

**Published:** 2021-12-22

**Authors:** Jonathan Mirault, Charlotte Leflaëc, Jonathan Grainger

**Affiliations:** Laboratoire de Psychologie Cognitive, CNRS & Aix-Marseille University, 3 place Victor Hugo, 13331 Marseille, France

**Keywords:** Reading, Grammatical decisions, Transposed words, Orthographic relatedness

## Abstract

In two on-line experiments (*N* = 386) we asked participants to make speeded grammatical decisions to a mixture of syntactically correct sentences and ungrammatical sequences of words. In Experiment 1, the ungrammatical sequences were formed by transposing two inner words in a correct sentence (e.g., *the brave daunt the wind* / *the daunt brave the wind*), and we manipulated the orthographic relatedness of the two transposed words (e.g., *the brave brace the wind* / *the brace brave the wind*). We found inhibitory effects of orthographic relatedness in decisions to both the correct sentences and the ungrammatical transposed-word sequences. In Experiment 2, we further investigated the impact of orthographic relatedness on transposed-word effects by including control ungrammatical sequences that were matched to the transposed-word sequences. We replicated the inhibitory effects of orthographic relatedness on both grammatical and ungrammatical decisions and found that transposed-word effects were not influenced by this factor. We conclude that orthographic relatedness across adjacent words impacts on processes involved in parallel word identification for sentence comprehension, but not on the association of word identities to positions in a sequence.

Effects of orthographic relatedness among words have been much investigated in single word recognition studies, where the orthographically related words are not actually physically present (e.g., Andrews, 1989; Grainger et al., 1989; see Grainger, 2018, for a review), but much less so in the context of sentence reading. Some studies manipulated the number of orthographic neighbors and the frequency of these words, as in single word recognition studies, but in a sentence reading context (e.g., Perea & Pollatsek, 1998; Pollatsek et al., 1999). In the present work, we examine the impact of orthographic relatedness across two words that are physically present in a sentence context, as opposed to the effects of virtual neighbors.

This has already been investigated in the seminal work of Paterson et al. (2009). In that study, Paterson and colleagues examined effects of orthographic relatedness across nonadjacent words during sentence reading. Participants read sentences for meaning while their eye movements were recorded. Half of the sentences included nonadjacent orthographically related words such as “There was a *blur* as the *blue* lights of the police car . . . ,” and eye gaze durations on the target word “blue” were compared with a condition with no orthographic relatedness (“There was a *gasp* as the *blue* lights of the police car . . .”). Processing of the target word “blue” was impaired (longer first fixation and gaze durations) in the related condition compared with the unrelated condition. Paterson et al. concluded that the lexical representation of the first word of an orthographically related pair remained activated and interfered during the processing of the second (target) word of the pair (an effect of lexical competition). In the present work we turn to examine effects of orthographic relatedness across *adjacent* words during sentence reading. This was motivated by the contradictory findings obtained in prior research, which we will now examine.

Effects of orthographic overlap across adjacent words during sentence reading have been investigated with the parafoveal preview paradigm using the boundary technique (Rayner, 1975) such that when fixating word *N*, the stimulus immediately to the right (the *N* + 1 preview) is either related or not to the upcoming target word, and as readers’ eyes move to position *N* + 1 the preview stimulus changes to become the target word (e.g., “The slight blue of the lights . . .” => “The slight blur of the lights . . . ,” where the target is the word “blur”). Orthographic relatedness was found to facilitate processing of the target word (i.e., shorter gaze durations) when the parafoveal preview is both a word and a nonword (Williams et al., 2006).

In more recent work, the same pattern of findings has been obtained using a parafoveal-on-foveal manipulation, such that when fixating word *N*, the word immediately to the right (*N* + 1) can be orthographically related to word *N* or not, and as readers’ gaze moves to position *N* + 1 the word at that location is changed to become a regular continuation of the sentence (e.g., “The slight blur blue the shape of . . .” => “The slight blur took the shape of . . . ,” where the target is the word “blur”). Orthographic relatedness has been found to facilitate processing of the target word (i.e., shorter gaze durations) when the parafoveal stimulus (*N* + 1) is both a word and a nonword (Angele et al., 2013; Dare & Shillcock, 2013; Inhoff et al., 2000; Mirault & Grainger, 2020; Snell et al., 2017). In line with these parafoveal-on-foveal facilitation effects are the findings obtained with the flankers task, where a central target word is flanked to the left and to the right be letters that are related or not to the target. Once again, orthographically related flankers were found to facilitate target word processing independently of whether they were words or nonwords (Snell et al., 2017).

These facilitatory effects of orthographically related parafoveal stimuli contrast with the interference found with nonadjacent orthographically related words during sentence reading (Paterson et al., 2009). What might be driving this discrepancy? Over and above the obvious fact that stimuli were nonadjacent in the only study so far to reveal inhibition, a condition that we will demonstrate is not necessary, here we propose and put to the test one possible explanation of this discrepancy. That is, that the inhibitory effects are driven by the goal to assign unique word identities to specific locations (necessary for sentence comprehension), whereas the facilitatory effects are driven by the parallel processing of words that appear at distinct spatial locations but where the assignment of word identities to different locations is not necessary for the task at hand. We further hypothesize that the association of word identities to different locations involves conscious identification of words and retrieval of the corresponding syntactic and semantic information (Snell & Grainger, 2019a; White et al., 2019).

One computational model of word identification and sentence reading—OB1-reader (Snell, van Leipsig, et al., 2018b)—provides the basis for a formal account of how this might occur. Orthographic facilitation would be driven by the spatial integration of orthographic information across adjacent stimuli, with this information being pooled into a single channel for parallel word processing. Here, we propose one key modification of OB1 such that words do not compete for identification within this single processing channel, but only compete for identification when associated with a specific location in a sequence of words, and crucially only when several words have to be identified, as is the case for sentence comprehension (see Fig. 1). The competition associated with multiple word identification would then have repercussions later on during sentence processing when the related nonidentified word is presented (Paterson et al., 2009)—a form of orthographic repetition blindness (Harris & Morris, 2000). In line with this logic is the fact that all demonstrations of facilitatory effects of orthographic overlap across adjacent words have been obtained using paradigms where the related nontarget word is not necessarily consciously identified (e.g., parafoveal-preview and parafovea-on-fovea manipulations using the boundary technique).

**Fig. 1:**
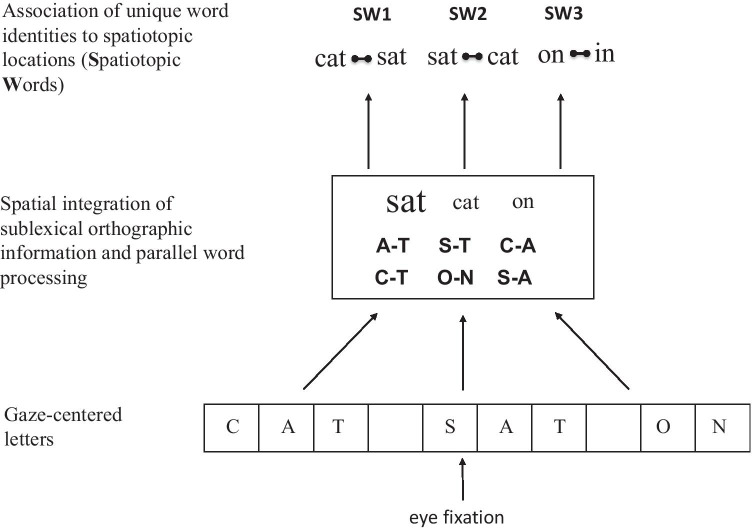
Modified version of the part of the architecture of OB1-reader (Snell, van Leipsig, et al., 2018b) concerning parallel word processing during reading. Information spanning multiple words processed by gaze-centered (location-specific) letter detectors is pooled into a single channel for location-invariant sublexical orthographic processing (via a bag-of-bigrams) and parallel word processing (bag-of-words). Relative activation levels of coactive words (illustrated by differences in size) is determined by acuity, crowding, spatial attention, and length-matching. Word identities then compete for their unique association with a given spatiotopic location along a line of text. Spatiotopic coordinates provide information about word-in-sentence position independently of eye fixation position. The key modification relative to OB1 is that lateral inhibition only operates at the level of spatiotopic words

In the present study, we test the prediction that the interfering effects of adjacent orthographically related words can be observed in a measure of global sentence processing difficulty. To do so we use the grammatical decision task, recently introduced as the sentence-level equivalent of the popular lexical decision task (Mirault et al., 2018; Mirault & Grainger, 2020). Participants made grammatical decisions to correct sentences containing adjacent orthographic neighbors (e.g., “the brave brace the wind”) and these were compared with decisions to matched sentences not containing orthographically related words (e.g., “the brave daunt the wind”).

Finally, in the present study we also examined the impact of orthographic relatedness on a new phenomenon revealed in research using the grammatical decision task: transposed-word effects (Mirault et al., 2018; Snell & Grainger, 2019b). Intuitively, one might expect orthographic relatedness to increase confusability in the association of words to locations, and therefore one is led to predict that transposed-word effects should be greater when the two transposed words are orthographic neighbors (an interaction effect). On the other hand, if (i) lexical competition is limited to a given location (see Fig. 1) and (ii) transposed-word effects are driven by a combination of noisy bottom-up position coding^1^ and top-down syntactic constraints (Snell & Grainger, 2019b) that function independently of orthographic relatedness, then we are led to predict that the effects should be additive with effects of orthographic relatedness.

## Experiment 1

### Methods

#### Participants

One hundred and ninety-two volunteers (85 females) participated in a 20-minute online experiment using their personal computers. The participants, ranged in age from 18 to 71 years (*M* = 22.26 years, *SD* = 10.27), and were naïve to the purpose of the experiment. They were informed prior to the beginning of the experiment that data would be collected anonymously.

#### Design and stimuli

We constructed 50 sentences in French, ranging in length from five to 10 words (*M* = seven words), including two adjacent words (a noun and a verb) that were neither the first nor the last word in the sentence. From these base sentences, we created a set of new sentences replacing the verb with another verb that was an orthographic neighbor of the noun (the two words differed by only one inner letter). These two sets of sentences define the relatedness factor (two adjacent orthographically related words or not). We further created ungrammatical transposed-word sequences by transposing the noun and the verb in the set of grammatically correct sentences. Relatedness was also manipulated in the transposed-word sequences in the same way as for the correct sentences. We performed two separate analyses for data pertaining to the correct sentences and for data for the ungrammatical sequences. Examples of the four types of word sequences are shown in Table 1 (in English, for convenience).

**Table 1:** Example of the different sequences of words tested in Experiment 1

Grammatical	related	the brave brace the wind
	unrelated	the brave daunt the wind
Transposed	related	the brace brave the wind
	unrelated	the daunt brave the wind

*Note*. Examples are in English, but the experiment was in French

#### Apparatus

The experiment was created with LabVanced (Finger et al., 2017) and we used the Prolific platform (Pallan & Schitter, 2018) to recruit participants.

#### Procedure

**Fig. 2:**
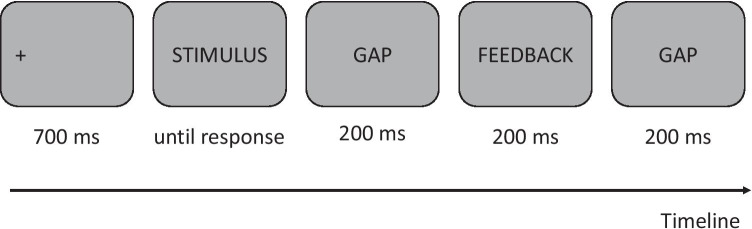
Procedure of one experimental trial

Placed in front of a computer screen, the participants had to click onscreen to accept to participate to the experiment. They were informed that the data would be anonymously recorded. Then, instructions were presented, and the participants had to press the space bar when they had read and understood the instructions in order to access the practice trials. These were composed of 12 trials that were representative of the conditions tested in the main experiment, but were not presented in the main experiment. At the end of the practice trials, participants were invited to press the space bar to start the main experiment which consisted of 200 trials. Each trial started with a fixation cross presented for 700 ms on the left edge of the upcoming sequence of words follows by a gap of 200 ms. Then the word sequence was presented, centered horizontally, and remained visible until participants’ response. Participants were instructed to press the right arrow of their computer keyboard if the sequence of words was grammatically correct or to press the left arrow otherwise. Finally, feedback was presented during 200 ms in the middle of the screen in the form of a green dot if the response was correct or a red cross if incorrect. A gap of 200 ms separated the feedback from the beginning of the following trial (see Fig. 2 for a summary of the procedure). The 50 items were displayed four times (in all four conditions), and the order of presentation was randomly determined for each participant. A pause was proposed after every 50 trials.

#### Analysis

We used linear mixed-effects models (LME) to analyze response times (RTs) and generalized (logistic) linear mixed-effects models (GLME) to analyze error rate, with participants and items as crossed random effects (Baayen et al., 2008; Barr et al., 2013). The models were fitted with the lmer (for LME) and the glmer (for GLME) functions from the lme4 package (Bates et al., 2015) in the R statistical computing environment (R Core Team, 2018). We report regression coefficients (*b*), standard errors (*SE*), and *t* values (for LME) or *z* values (for GLME). Fixed effects were deemed reliable if |*t*| or |*z*| > 1.96 (Baayen 2008). RTs were log transformed prior to analysis in order to normalize the distribution. We used the maximal random structure model that converged (Barr et al., 2013), and this included by-participant and by-item random intercepts in all analyses we report.

### Results

Prior to analysis, we excluded 19 participants with an average accuracy of less than 75%. Then, we deleted 6.35% of the trials with RTs less than 100 ms or greater than 10,000 ms. The remaining dataset was composed of 34,402 observations, which largely exceeds the recommendation of Brysbaert and Stevens (2018). We further estimated power using the SIMR package in the R environment (Green & Macleod, 2016). With more than 1,000 simulations we attained an estimated statistical power of 97.40% (95% CI = 2.08) for effects of relatedness in the correct sentences and 98.90% (95% CI = 1.41) for effects of relatedness in the ungrammatical transposed-word condition.

#### Response time

Prior to analysis, we further excluded trials with incorrect responses (7.77%) and values lying beyond 2.5 standard deviations from the grand mean (2.21%). The remaining dataset was composed of 29,223 observations. We observed a significant effect of relatedness in responses to the grammatically correct sentences (*b* = 0.01, *SE* = 0.004, *t* = 3.93), with participants taking longer to respond when there were two orthographically related words in the sentence. There was also a significant effect of relatedness in responses to the ungrammatical transposed-word sequences (*b* = 0.02, *SE* = 0.004, *t* = 4.61), with longer RTs when the transposed words were orthographically related. Condition means are reported in Table 2.

**Table 2:** Average RT (in ms) per experimental condition in Experiment 1

	Related	Unrelated	Effect
Grammatical	1,870 (12.90)	1,816 (12.97)	−54
Transposed	2,026 (15.57)	1,939 (14.93)	−87

*Note*. Values between parentheses are within-participant 95% CIs (Cousineau, 2005)

#### Error rate

We observed a significant effect of Relatedness in responses to the correct sentences (*b* = 0.48, *SE* = 0.07, *z* = 6.24), with participants making more errors when there were two orthographically related words in the sentence. There was also a significant effect of relatedness in responses to the ungrammatical transposed-word sequences (*b* = 0.72, *SE* = 0.13, *z* = 5.50), with more errors when the transposed words were orthographically related. Condition means are reported in Table 3.

**Table 3:** Average error rates (in %) per experimental condition in Experiment 1

	Related	Unrelated	Effect
Grammatical	7.70 (0.56)	5.30 (0.54)	−2.40
Transposed	11.07 (0.66)	7.09 (0.64)	−3.98

*Note*. Values between parentheses are within-participant 95% CIs (Cousineau, 2005)

### Discussion

Experiment 1 found the predicted interfering effects of orthographic relatedness when making grammatical decisions to sequences of words. Grammatical decisions were harder to make (more errors and longer RTs) to correct sentences when the sentences contained two adjacent words that were orthographic neighbors. The fact that these effects were found when the two orthographically related words were adjacent allows us to reject a simple account of prior findings according to which non-adjacency is a necessary condition for obtaining inhibitory effects.

Experiment 1 also tested for effects of orthographic relatedness on grammatical decisions made to transposed-word sequences (i.e., where the ungrammaticality was created by transposing two words in a correct sentence). Ungrammatical decisions were also harder to make (more errors and longer RTs) when the transposed words were orthographic neighbors. This could be taken as evidence that orthographic similarity led to increased confusability in the process of associating word identities to positions in a sequence of words. However, Experiment 1 lacked an appropriate control condition for measuring the impact of orthographic relatedness on transposed-word effects. That is, there were no ungrammatical sequences containing orthographically related words that could not be resolved into a grammatically correct sequence by transposing the two critical words. Hence, in Experiment 2, we therefore compared the effects of orthographic relatedness on transposed-word effects against the effects obtained with a new set of ungrammatical sequences that were not formed by transposing two words in a correct sentence (i.e., a correct sentence could not be generated by transposing any two words in these ungrammatical sequences). This enabled a proper test of the impact of orthographic relatedness on word position coding.

## Experiment 2

### Methods

#### Participants

One hundred and ninety-four volunteers (89 females) participated in a 20-minute online experiment using their personal computers. We estimated power for Experiment 2 a priori using the results of Experiment 1 with SIMR (Green & Macleod, 2016). This gave an estimated statistical power of 89.10% (95% CI = 3.96) for observing an effect of Relatedness in the transposed-word condition for this number of participants. The participants, ranged in age from 18 to 72 years (*M* = 28.67, *SD* = 9.60), and were naïve as to the purpose of the experiment. They were informed prior to the beginning of the experiment that data would be collected anonymously.

#### Design and stimuli

We constructed 100 sentences in French that ranged in length from four to 10 words (*M* = 7.06 words, *SD* = 1.09). For 50 of these sentences, two sentence-internal adjacent words were orthographically related (i.e., they differed by only one letter, which was neither the first nor the last letter). These two sets of sentences represent the two levels of the relatedness factor. From these 100 base sentences, we created 200 ungrammatical sequences. Half of these were formed by transposing two adjacent words in the correct sentences. The other half were formed by first transposing the two same two adjacent words and then replacing one other sentence-internal word with a different word of the same length such that transposing any two words would not generate a correct sentence (see Table 4). This formed the two levels of the transposition factor—transposed word versus control (see Appendix for a list of the stimuli). We added 100 correct sentences (50 with orthographically related adjacent words, 50 without) for the purpose of the grammatical decision task. The key manipulation here was on the ungrammatical sequences following a 2 × 2 factorial design with the factors relatedness and transposition. The effect of relatedness on the grammatically correct sentences was analyzed separately.

**Table 4:** Examples of how the critical ungrammatical word sequences (transposed and control) were created in Experiment 2

Base sentence	*related*	the brave brace the wind
	*unrelated*	our cats are very tired
Transposed	*related*	the brace brave the wind
	*unrelated*	our are cats very tired
Control	*related*	the brace brave not wind
	*unrelated*	our are cats long tired

*Note.* Examples are in English, but the experiment was in French. The base sentences were not shown in the experiment, but a different set of sentences with or without related words was used for the purpose of the grammatical decision task

#### Procedure

The procedure was the same as in Experiment 1 (see Fig. 2).

#### Analysis

The same analyses as for Experiment 1 were performed but focusing this time on the critical ungrammatical sequences. The results concerning the grammatical sequences are also reported for comparison with Experiment 1, but were analyzed separately.

### Results

Prior to analysis, we excluded four participants with an average performance less than 75% correct. Then we deleted 5.83% of trials with RTs below 100 ms or above 4,000 ms. The remaining dataset was composed of 35,593 observations, a number that largely exceeds the recommendation of Brysbaert and Stevens (2018).

#### Response time

Prior to analysis, we further excluded trials on which there was an incorrect response (7.93%) and RT values lying beyond 2.5 standard deviations from the grand mean (2.67%). The remaining dataset was composed of 31,892 observations. Condition means are reported in Table 5. We found a significant effect of relatedness (*b* = 0.02, *SE* = 0.009, *t* = 2.74), with longer RTs in when the sequences contained two orthographically related words. There was also a significant effect of transposition (*b* = 0.01, *SE* = 0.005, *t* = 2.09), with longer RTs in the transposed-word condition compared with the control condition. The interaction was not significant (*b* = 0.004, *SE* = 0.007, *t* = 0.66). There was also a significant effect of relatedness on RTs to the correct sentences (*b* = 0.06, *SE* = 0.01, *t* = 6.35).

**Table 5:** Average RTs (in ms) per experimental condition in Experiment 2

	Related	Unrelated	Effect
Transposed	1,943 (21.12)	1,832 (21.00)	−111
Control	1,919 (20.57)	1,809 (19.19)	−110
Grammatical	1,806 (11.03)	1,544 (14.43)	−262

*Note*. Values between parentheses are within-participant 95% CIs (Cousineau, 2005)

#### Error rate

Condition means are shown in Table 6. The effect of relatedness was not significant in decisions made to the ungrammatical sequences (*b* = 0.37, *SE* = 0.23, *z* = 1.55). The effect of Transposition was significant (*b* = 1.26, *SE* = 0.10, *z* = 12.29), such that participants made more errors in the transposed-word condition compared to the control condition. The Relatedness × Transposition interaction was not significant (*b* = 0.11, *SE* = 0.12, *z* = 0.91). Finally, there was a significant effect of relatedness on decisions made to the correct sentences (*b* = 1.91, *SE* = 0.31, *z* = 6.06).

**Table 6:** Average error rates (in %) per experimental condition in Experiment 2

	Related	Unrelated	Effect
Transposed	15.10 (1.63)	12.02 (1.60)	−3.08
Control	6.95 (1.19)	5.58 (0.97)	−1.37
Grammatical	9.24 (0.89)	3.21 (0.31)	−6.03

*Note.* Values between parentheses are within-participant 95% CIs (Cousineau, 2005)

### Discussion

The effects of Relatedness found in Experiment 1 were successfully replicated in Experiment 2, although limited to RTs in decisions made to the ungrammatical sequences. Crucially, we further observed that the size of transposed-word effects was not influenced by orthographic relatedness. The negative impact of relatedness on RTs was practically equivalent (111 ms and 110 ms) in the transposed-word sequences and the corresponding control sequences (see Table 5).

## General discussion

The present study was motivated by contradictory results concerning the effects of orthographically related words (orthographic neighbors) during sentence reading when the target word and its orthographic neighbor are physically present. When the two words are separated by other words, then inhibitory effects of orthographic relatedness have been found (Paterson et al., 2009). On the other hand, when the two related words are adjacent, as is the case in parafoveal-preview (Williams et al., 2006) and parafovea-on-fovea studies (Snell et al., 2017), then the effects are facilitatory. In order to account for these discrepant findings we proposed a modification of the architecture of the OB1-reader model (Snell, van Leipsig, et al., 2018b) that introduces a key distinction between parallel word processing without competition in the central processing channel, and unique word identification with competition when multiple words are associated with a specific location along a line of text (see Fig. 1). We then predicted that two adjacent orthographically similar words would compete when both of these words have to be identified for successful sentence comprehension (which is not the case in parafoveal preview and parafovea-on-fovea studies).

In line with this prediction, we found that orthographic relatedness across adjacent words made it harder to make grammatical decisions to both grammatical and ungrammatical word sequences. The fact that we found effects in the same direction with both types of decision is important since it shows that participants were not just judging word sequences containing orthographically related words to be less grammatical. This provides a replication of the findings of Paterson et al. (2009), this time with adjacent words and with a measure of global sentence reading time (grammatical decisions). Our findings therefore allow us to reject the adjacency account of the divergent findings found in prior research, where inhibition was found with nonadjacent orthographically related words (Paterson et al., 2009) and facilitation with adjacent orthographically related words (Snell et al., 2017). Our findings suggest that multiple word identification is the key to observing inhibitory effects of orthographic neighbors, and that the central processing channel in OB1-reader (Snell, van Leipsig, et al., 2018b) outputs probabilities for several word identities simultaneously in the absence of competitive interactions between these words. It is only when these words are associated with different locations along a line of text that competitive processes kick in.

However, when several words are presented at the same location, as in priming studies, then these words compete for identification at that location (e.g., Davis & Lupker, 2006; Segui & Grainger, 1990). The same reasoning holds for effects of virtual neighbors in single word recognition studies (e.g., Grainger et al., 1989), word recognition in the flankers task (Meade et al., 2021), and during sentence reading (Williams et al., 2006), where multiple words, although not physically present, compete for identification at a given location. In line with this reasoning is that fact that single word presentation studies typically find facilitatory effects of orthographic neighbors when superficial response strategies, as opposed to word identification, are encouraged (e.g., Andrews, 1989; Grainger & Jacobs, 1996). In further support of the above reasoning, Snell, Bertrand, and Grainger (2018a) compared effects of orthographically related words with a priming manipulation and a flanker manipulation. Testing the same set of words with both procedures, Snell, Bertrand, and Grainger (2018a) reported the standard inhibitory priming effect with orthographically related prime words relative to unrelated prime words, and, on the contrary, a facilitatory effect of orthographically related flanker words.

Experiment 2 of the present study provided a further investigation of transposed-word effects, this time in a context where the adjacent transposed-words could be orthographically similar or not. Effects of orthographic relatedness were compared across the ungrammatical transposed-word sequences and the matched ungrammatical sequences that could not be resolved into a correct sentence by transposing two words. We found statistically equivalent inhibitory effects of orthographic relatedness in RTs and error rates to both types of ungrammatical sequence.

One might have expected orthographically similar words to be more confusable and that this increased confusability would lead to greater positional uncertainty and greater transposed-word effects. This is not what we observed. This pattern of results therefore points to independent processes that assign word identities to locations on the one hand, and that govern competitive interactions among words assigned to the same location on the other. Once a word is associated with a specific position in the sequence of words being read, with a certain amount of positional noise, it is only after this process of word–position association that competition between orthographically similar words starts to operate. Thus, when reading the sequence of words “the brace brave the wind,” the association of the words “brace” and “brave” to Positions 2 and 3, respectively, is subject to bottom-up positional noise and top-down syntactic constraints, but independently of how orthographically similar the two words are. It is this process that gives rise to transposed-word effects. Once these words have been associated with a given position, with a certain probability, then position-specific competitive processes enter the scene. Two orthographically similar adjacent words will compete for identification at a given position in the sequence, because they will also be associated, albeit with a lower probability, with the adjacent position. Finally, given that length information is hypothesized to guide the allocation of word identities to positions, it would be interesting to investigate in future research whether or not other types of visual information, such as letter case, might help reduce positional noise in this process.

### Conclusions

In two experiments, we examined the impact of orthographic relatedness across adjacent words on the ease with which participants can make grammatical decisions to sequences of words. We found inhibitory effects of orthographic relatedness in decisions made to both grammatical and ungrammatical word sequences. Crucially, the difficulty in deciding that transposed-word sequences were ungrammatical was not affected by orthographic relatedness. We conclude that transposed-word effects are driven by processes that operated independently of whether or not the two words are orthographically related, and that the negative impact of orthographic relatedness reflects competition for identification at a given position in a sequence of words. That is, words compete for a given slot in space during reading in the same way that words compete for a given slot in time during spoken language comprehension.

## Footnotes

1 Concerning the precise nature of the positional noise that affects the association of word identities to word positions, we assume that this takes the form of positional uncertainty as in Gomez et al.’s (2008) model of letter position coding, this time applied to encoding word-in-sentence order rather than letter-in-word order. This therefore differs from the letter-order encoding mechanism shown in Fig. 1, where a relative-position coding scheme is applied.

## References

[CR1] Andrews S (1989). Frequency and neighborhood effects on lexical access: Activation or search?. Journal of Experimental Psychology: Learning, Memory, and Cognition.

[CR2] Angele B, Tran R, Rayner K (2013). Parafoveal–foveal overlap can facilitate ongoing word identification during reading: Evidence from eye movements. Journal of Experimental Psychology: Human Perception and Performance.

[CR3] Baayen R (2008) Analyzing linguistic data: a practical introduction to statistics. Cambridge University Press

[CR4] Baayen RH, Davidson DJ, Bates DM (2008). Mixed-effects modeling with crossed random effects for subjects and items. Journal of Memory and Language.

[CR5] Barr DJ, Levy R, Scheepers C, Tily HJ (2013). Random effects structure for confirmatory hypothesis testing: Keep it maximal. Journal of Memory and Language.

[CR6] Bates DJ, Maechler M, Bolker B, Walker S (2015). Fitting linear mixed-effects models using lme4. Journal of Statistical Software.

[CR7] Brysbaert, M., & Stevens, M. (2018). Power analysis and effect size in mixed effects models: A tutorial. *Journal of Cognition*, *1*(1). 10.5334/joc.10 10.5334/joc.10 PMC6646942 31517183

[CR8] Cousineau D (2005). Confidence intervals in within-subject designs: A simpler solution to Loftus and Masson’s method. Tutorials in Quantitative Methods for Psychology.

[CR9] Dare N, Shillcock R (2013). Serial and parallel processing in reading: Investigating the effects of parafoveal orthographic information on nonisolated word recognition. Quarterly Journal of Experimental Psychology.

[CR10] Davis CJ, Lupker SJ (2006). Masked inhibitory priming in English: Evidence for lexical inhibition. Journal of Experimental Psychology: Human Perception and Performance.

[CR11] Finger, H., Goeke, C., Diekamp, D., Standvoß, K., & König, P. (2017). *LabVanced: A unified JavaScript framework for online studies*. Paper presented at the 2017 International Conference on Computational Social Science IC2S2, Cologne, Germany.

[CR12] Gomez P, Ratcliff R, Perea M (2008). The overlap model: A model of letter position coding. Psychological Review.

[CR13] Grainger J (2018). Orthographic processing: A ‘mid-level’ vision of reading: The 44th Sir Frederic Bartlett Lecture. Quarterly Journal of Experimental Psychology.

[CR14] Grainger J, Jacobs AM (1996). Orthographic processing in visual word recognition: a multiple read-out model. Psychological Review.

[CR15] Grainger J, O’regan JK, Jacobs AM, Segui J (1989). On the role of competing word units in visual word recognition: The neighborhood frequency effect. Perception & Psychophysics.

[CR16] Green P, MacLeod CJ (2016) SIMR: an R package for power analysis of generalized linear mixed models by simulation. Methods Ecol Evol 7:493–498

[CR17] Harris CL, Morris AL (2000). Orthographic repetition blindness. The Quarterly Journal of Experimental Psychology Section A.

[CR18] Inhoff AW, Starr M, Shindler KL (2000). Is the processing of words during eye fixations in reading strictly serial?. Perception & Psychophysics.

[CR19] Meade G, Grainger J, Declerck M (2021). Friend or foe? Flankers reverse the direction of orthographic neighborhood effects. Language, Cognition, and Neuroscience.

[CR20] Mirault J, Grainger J (2020). On the time it takes to judge grammaticality. Quarterly Journal of Experimental Psychology.

[CR21] Mirault J, Snell J, Grainger J (2018). You that read wrong again! A transposed-word effect in grammaticality judgments. Psychological Science.

[CR22] Pallan S, Schitter C (2018). Prolific.ac—A subject pool for online experiments. Journal of Behavioral and Experimental Finance.

[CR23] Paterson KB, Liversedge SP, Davis CJ (2009). Inhibitory neighbor priming effects in eye movements during reading. Psychonomic Bulletin & Review.

[CR24] Perea M, Pollatsek A (1998). The effects of neighborhood frequency in reading and lexical decision. Journal of Experimental Psychology: Human Perception and Performance.

[CR25] Pollatsek A, Perea M, Binder KS (1999). The effects of “neighborhood size” in reading and lexical decision. Journal of Experimental Psychology: Human Perception and Performance.

[CR26] R Core Team (2018). R: A language and environment for statistical computing [Computer software].

[CR27] Rayner K (1975). The perceptual span and peripheral cues in reading. Cognitive psychology.

[CR28] Segui J, Grainger J (1990). Priming word recognition with orthographic neighbors: Effects of relative prime–target frequency. Journal of Experimental Psychology: Human Perception and Performance.

[CR29] Snell J, Grainger J (2019). Readers are parallel processors. Trends in Cognitive Sciences.

[CR30] Snell J, Grainger J (2019). Word position coding in reading is noisy. Psychonomic Bulletin & Review.

[CR31] Snell J, Vitu F, Grainger J (2017). Integration of parafoveal orthographic information during foveal word reading: Beyond the sub-lexical level?. Quarterly Journal of Experimental Psychology.

[CR32] Snell J, Bertrand D, Grainger J (2018). Parafoveal letter-position coding in reading. Memory & Cognition.

[CR33] Snell J, van Leipsig S, Grainger J, Meeter M (2018). OB1-reader: A model of word recognition and eye movements in text reading. Psychological Review.

[CR34] White AL, Boynton GM, Yeatman JD (2019). You can’t recognize two words simultaneously. Trends in Cognitive Sciences.

[CR35] Williams CC, Perea M, Pollatsek A, Rayner K (2006). Previewing the neighborhood: The role of orthographic neighbors as parafoveal previews in reading. Journal of Experimental Psychology: Human Perception and Performance.

